# The phenotypic variations of multi-locus imprinting disturbances associated with maternal-effect variants of *NLRP5* range from overt imprinting disorder to apparently healthy phenotype

**DOI:** 10.1186/s13148-019-0760-8

**Published:** 2019-12-11

**Authors:** Angela Sparago, Ankit Verma, Maria Grazia Patricelli, Laura Pignata, Silvia Russo, Luciano Calzari, Naomi De Francesco, Rosita Del Prete, Orazio Palumbo, Massimo Carella, Deborah J. G. Mackay, Faisal I. Rezwan, Claudia Angelini, Flavia Cerrato, Maria Vittoria Cubellis, Andrea Riccio

**Affiliations:** 1 Department of Environmental, Biological and Pharmaceutical Sciences and Technologies (DiSTABiF), Università degli Studi della Campania “Luigi Vanvitelli”, Caserta, Italy; 2 Institute of Genetics and Biophysics (IGB) “Adriano Buzzati-Traverso”, Consiglio Nazionale delle Ricerche (CNR), Naples, Italy; 3 Molecular Biology and Citogenetics, IRCCS San Raffaele Scientific Institute, Milan, Italy; 4 Medical Cytogenetics and Molecular Genetics Laboratory, Centro di Ricerche e Tecnologie Biomediche IRCCS, Istituto Auxologico Italiano, Milan, Italy; 5 Medical Genetics Unit, IRCCS Casa Sollievo della Sofferenza, San Giovanni Rotondo, FG Italy; 6 Faculty of Medicine, University of Southampton, Southampton, UK; 7 Institute for Applied Mathematics “Mauro Picone” (IAC), Consiglio Nazionale delle Ricerche (CNR), Napoli, Italy; 8 Department of Biology, Università degli Studi di Napoli “Federico II”, Napoli, Italy

**Keywords:** Multi-locus imprinting disturbances, NLRP5, Beckwith-Wiedemann syndrome, Genomic imprinting, DNA-methylation, Maternal-effect variants

## Abstract

**Background:**

A subset of individuals affected by imprinting disorders displays multi-locus imprinting disturbances (MLID). MLID has been associated with maternal-effect variants that alter the maintenance of methylation at germline-derived differentially methylated regions (gDMRs) in early embryogenesis. Pedigrees of individuals with MLID also include siblings with healthy phenotype. However, it is unknown if these healthy individuals have MLID themselves or if their methylation patterns differ from those associated with imprinting disorders, and in general, if MLID affects the clinical phenotype.

**Methods:**

We have investigated gDMR methylation by locus-specific and whole-genome analyses in a family with multiple pregnancy losses, a child with Beckwith-Wiedemann syndrome (BWS) and a further child with no clinical diagnosis of imprinting disorder or other pathologies.

**Results:**

We detected MLID with different methylation profiles in the BWS-affected and healthy siblings. Whole-exome sequencing demonstrated the presence of novel loss-of-function variants of *NLRP5* in compound heterozygosity in the mother. The methylation profiles of the two siblings were compared with those of other cases with MLID and control groups by principal component analysis and unsupervised hierarchical clustering, but while their patterns were clearly separated from those of controls, we were unable to cluster those associated with specific clinical phenotypes among the MLID cases.

**Conclusion:**

The identification of two novel maternal-effect variants of *NLRP5* associated with poly-abortivity and MLID adds further evidence to the role of this gene in the maintenance of genomic imprinting in early embryos. Furthermore, our results demonstrate that within these pedigrees, MLID can also be present in the progeny with healthy phenotype, indicating that some sort of compensation occurs between altered imprinted loci in these individuals. The analysis of larger cohorts of patients with MLID is needed to formulate more accurate epigenotype-phenotype correlations.

## Introduction

Imprinting disorders are a clinically heterogeneous group of diseases characterized by defective expression associated with genetic or epigenetic abnormalities of imprinted genes [1]. DNA methylation abnormalities in imprinting disorders typically affect a germline-derived differentially methylated region (gDMR) that regulates the gamete-of-origin-specific expression of a cluster of imprinted genes [1]. For example, most of the individuals affected by Beckwith-Wiedemann syndrome (BWS) have altered DNA methylation of either the *H19-IGF2*:IG-DMR (also known as IC1) or the *KCNQ1OT1*:TSS-DMR (also known as IC2) both located on chromosome 11p15.5 that regulate two independent gene clusters [2]. A subgroup of patients, however, exhibits multi-locus imprinting disturbances (MLID) that potentially alter the expression of multiple imprinted gene clusters [3, 4]. The percentage of patients with MLID depends on type of imprinting disorder and method of methylation analysis [3]. Individuals with MLID usually present with clinical features characteristic of one imprinting disorder, most frequently BWS, Silver-Russell syndrome (SRS), and transient neonatal diabetes mellitus (TNDM), but some cases show complex or atypical phenotypes, possibly reflecting the loci and tissues affected with the mosaic epigenetic abnormalities [5, 6]. However, due to the limited number of patients identified with MLID, its mosaic nature, the complexity of genome-wide methylation analysis and often lack of careful clinical re-examination, and epigenotype-phenotype correlations are still unclear.

Variants affecting genes expressed either in the early embryo or oocyte have been associated with MLID [1]. The best example of the formers is recessive variants of ZFP57 that have been found in individuals affected by TNDM [7]. In the mouse, ZFP57 has been demonstrated to interact with methylated gDMRs and prevent their demethylation during early embryogenesis [8, 9]. Concerning the oocyte factors, either recessive or dominant variants of maternal-effect genes coding for components of the maternal sub-cortical complex (SCMC) have been reported in women with reproductive problems including multiple pregnancy losses and offspring with clinical features typical of imprinting disorders and/or developmental delay and behavioral problems [5, 6, 10, 11]. In particular, mutations in the NOD-like receptor family pyrin domain containing 5 gene (NLRP5) have been found in five mothers of individuals affected by BWS, SRS, or atypical imprinting disorders including cases with idiopathic developmental delay and autism, and families affected by infertility and reproductive wastage [6, 10, 11]. Loss of function (nonsense, frameshift, splicing) *NLRP5* variants are very rare in the general population and are reported in the ExAC database (http://exac.broadinstitute.org/) only in heterozygosity with an allele frequency of 0.0004. Missense variants predicted to be damaging have higher allele frequencies [6]. However, it should be considered that these variants are predicted to affect fertility and/or health of the progeny and these data are not recorded in the databases.

**Fig. 1:**
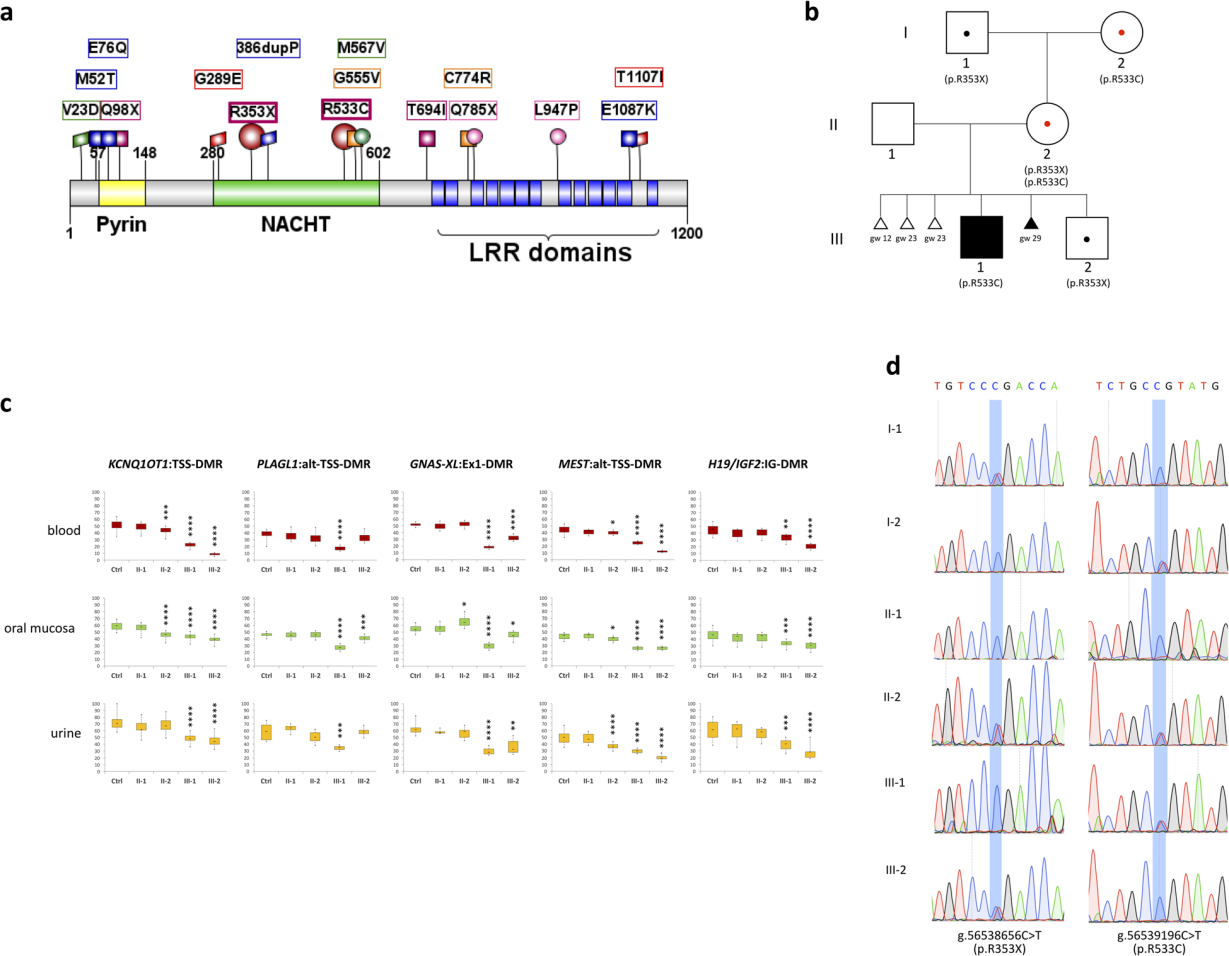
Novel *NLRP5* variants in a familial case of MLID with phenotypically discordant siblings. **a** Domain structure of human NLRP5 depicting the position of known variants [6, 10, 11], along with the two novel variants described in the present study (indicated by red circles). **b** Family pedigree and corresponding NLRP5 mutations. Black-filled symbols represent individuals with evident BWS features: proband (III-1) and fetus with omphalocele. Carriers of *NLRP5* variants are indicated by symbols with central dot (red in case of maternal carriers). Weeks of gestation are indicated for the four aborted fetuses. **c** Boxplot showing DNA methylation analysis of four maternal gDMRs (*KCNQ1OT1*, *PLAGL1*, GNAS, and *MEST*) and one paternal gDMR (*H19*), as measured by bisulphite pyrosequencing in three different tissue types. Primers used in PCR and sequencing have been checked for specificity of the assay. For each region, 6-12 CpGs have been tested and distribution of their percentage of methylation has been represented as boxplot. Data are a mean between at least two independent PCR and pyrosequencing experiments. Controls include 4–6 normal individuals. *p* values have been calculated by two-tailed Student’s *t* test (*p ≤ 0.05; ** *p* ≤ 0.01; ***p ≤ 0.001; ****p ≤ 0.0001). **d** Validation of *NLRP5* variants by Sanger sequencing and their segregation in the family. Variants are highlighted with a blue-shaded stripe and their genomic positions are indicated below the chromatogram (chr19, GRCh37/hg19). Note that the maternal grandmother (I-2) was heterozygous for the missense variant while the mother (II-2) was compound heterozygous for both the missense and nonsense variants

It is unknown how NLRP5 affects DNA methylation, but the missense variants associated with disease cluster in the pyrin, nucleotide-binding domain (NACHT), and leucine-rich repeat (LRR) domains (Fig. 1a), indicating a role of these domains for this function [12]. In other NLR proteins involved in inflammatory response (e.g., NLRP3), the pyrin domain is required for binding adaptor proteins, the NACHT domain for oligomerization, and the LRR domain for ligand binding [12]. Besides DNA methylation, NLRP5 is also controlling mitochondrial localization and activity as well as endoplasmic reticulum distribution and Ca++ homeostasis, and its downregulation has been associated with decreased levels of histone H3K9 trimethylation in mouse oocytes [13–15].

*NLRP5* and the other maternal-effect genes associated with MLID are highly expressed in oocytes, but their mutation affects early embryo development [1]. The mosaic methylation of both maternal and paternal gDMRs demonstrated in the offspring of carrier mothers suggests that imprinting maintenance in early embryo is impaired [1]. On the other hand, biallelic loss of function variants of other SCMC members, such as *NLRP7* and *KHDC3L*, are associated with lack of maternal imprinting establishment in oocytes and biparental hydatidiform mole [1].

In the case of maternal-effect variants, the recurrence risk after an affected pregnancy can be up to 100% [1]. Intriguingly, healthy siblings of patients with MLID have been reported in some pedigrees [5, 6]. However, methylation has not been investigated in these individuals so far, and it is unknown if the differential health status of the siblings is caused by different epigenetic profiles. This is particularly relevant, because some imprinting alterations are associated with cancer development [16, 17]. Inactivating these maternal-effect genes in the mouse has not been particularly instructive on the mechanism of MLID, because they either lead to very early embryo demise or cause limited imprinting alterations in the progeny [18, 19]. Recently, the use of arrays for assessing genome-wide methylation has increased the number of loci associated with MLID and improved its molecular diagnosis [20].

Here, we describe the genome-wide methylation profiles of two siblings with MLID, one presenting with overt BWS and the other with healthy phenotype, and whose mother has a history of multiple miscarriages and is compound heterozygous for loss of function variants of *NLRP5*.

## Results and discussion

The proband (III-1, Fig. 1b) is the first live-born child of a non-consanguineous healthy couple. BWS was diagnosed at birth because of the presence of typical features of this disease (score = 9) [2]. He also presented with atypical characteristics, such as low birth weight (< 0.4th centile), hypocalcemia, facial dysmorphism, and a slight cognitive delay. A younger brother (III-2) showed normal development and no features of BWS or other imprinting disorders at birth and during childhood (up to 10 years), apart from a slight facial asymmetry and placentomegaly during gestation. The couple also had four miscarriages, of which two displayed placentomegaly and one had exomphalos (Fig. 1b). Molecular confirmation of BWS was obtained in the proband by COBRA, which demonstrated loss of methylation (LOM) at the *KCNQ1OT1*:TSS-DMR (also known as IC2) in peripheral blood leukocytes (PBL, Additional file 1: Figure S1), the most common molecular defect of BWS [2]. Further investigation of the methylation status of five gDMRs by pyrosequencing demonstrated *KCNQ1OT1* LOM and MLID in III-1, but surprisingly also in III-2 (Fig. 1c). The methylation pattern was consistent in the DNAs extracted from three different tissues: PBL, oral mucosa (buccal brush), and cells derived from urine. The methylation level was lower than controls at *KCNQ1OT1*, *PLAGL1*, *GNAS*, *MEST*, and *H19/IGF2* in III-1, and at *KCNQ1OT1*, *GNAS*, *MEST*, and *H19/IGF2* in III-2 (see Fig. 1c for details on methylation levels). Notably, three of these loci including *KCNQ1OT1* were more hypomethylated in III-2 than in III-1 (*KCNQ1OT1* 31% versus 38%, *MEST* 20% versus 27%, *H19/IGF2* 26% versus 35%, as an average of the three tissues). Notably, a slight hypomethylation of *KCNQ1OT1* and *MEST* DMRs was also observed in II-2 by pyrosequencing (Fig. 1c). Consistent with *KCNQ1OT1* hypomethylation, the mRNA level of *CDKN1C* (an imprinted gene controlled by this DMR) that was detected in the oral mucosa of II-2, III-1, and III-2 was lower than that of control individuals (Additional file 2: Figure S2). Therefore, after the result of the molecular analysis, the phenotypes of III-2 and II-2 should be classified within the BWS spectrum (BWSp) [2].

Prompted by the familial occurrence of MLID and its association with multiple pregnancy loss, we looked for maternal-effect variants that could be responsible for the imprinting abnormalities in this family. The trio II-1, II-2, and III-1 was analyzed by whole-exome sequencing. Variants filtered as described in the methods were searched to find those occurring in genes highly expressed in oocytes [21]. We identified two rare variants of *NLRP5* in compound heterozygosity in II-2, the stop-gain R353X, and the missense R533C (Fig. 1a). Both variants occur in the NACHT domain [12]. The missense variant R533C is non-conservative, predicted as deleterious by PolyPhen2 [22], reported with a frequency of 0.00001660 in ExaC (http://exac.broadinstitute.org/), [23] and never observed in homozygosity. By Sanger sequencing (Fig. 1d), we confirmed the presence of these mutations in II-2 and demonstrated that she has inherited the nonsense variant from her father (I-1) and the missense variant from her mother (I-2), and transmitted the missense variant to III-1 and the nonsense variant to III-2. So, the segregation of the *NLRP5* variants in this family is consistent with that of maternal-effect mutations resulting in MLID in the progeny. In order to exclude other possible modalities of inheritance, we looked if any rare variant was present in the proband in homozygosity, hemizygosity, or compound heterozygosity. Rare variants present on both parental alleles were identified in only one gene (CEACAM20), but they were predicted to be benign by PolyPhen2 [22]. Moreover, single-nucleotide polymorphism (SNP) array analysis excluded any relevant copy-number variation or uniparental disomy in this family.

**Fig. 2:**
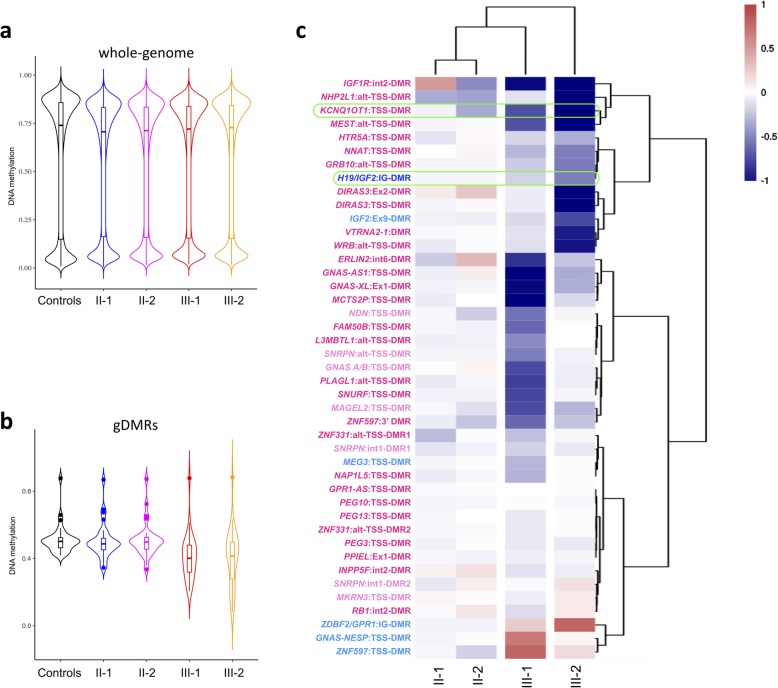
Characterization of DNA methylation in the family under study by genome-wide array analysis. **a** Violin plots showing distribution of mean CpG methylation level of the whole genome. **b** Violin plots showing distribution of mean CpG methylation level of 31 gDMRs (number of probes overlapped = 541) demonstrating significant differences in III-1 and III-2 (Additional file 3: Table S1). **c** Heatmap showing hierarchical clustering of imprinted DMR methylation levels for the parents (II-1 and II-2) and the two siblings (III-1 and III-2) normalized with six control individuals. Clustering is based on CpG methylation levels of 755 probes overlapping with 43 imprinted DMRs, containing at least three informative CpGs. Maternally methylated germline DMRs are in dark pink; maternally methylated secondary DMRs are in light pink. Paternally methylated germline DMRs are in dark blue; paternally methylated secondary DMRs are in light blue. The *KCNQ1OT1*:TSS-DMR and the *H19/IGF2*:IG-DMR diagnostic of BWS are highlighted in green

To better characterize the differences in the DNA methylation profiles of the two siblings, a genome-wide array analysis was performed on PBL DNAs of the two siblings, their parents, and six control individuals. After quality control filtering, methylation data for ~ 760,000 CpG sites for each sample were obtained. The results showed that, at the global level, both siblings have a general pattern of DNA methylation reminiscent of that of control individuals, without noticeable widespread methylation defects (Fig. 2a, Additional file 3: Table S1). By contrast, at gDMRs, both siblings showed lower average methylation than controls (median 40% for III-1 and 41% for III-2 versus 50%), indicating a selective effect on imprinted regions (Fig. 2b, Additional file 3: Table S1). The heatmap of Fig. 2c indicates the methylation level of all known imprinted DMRs covered by at least three informative CpGs [24]. The methylation levels of many imprinted loci in both III-1 and III-2 were different from those of their parents and controls. However, different profiles were evident in the two siblings. In particular, *MEG3*, *SNRPN/SNURF*, *PLAGL1*, *FAM50B*, and *GNAS* were more hypomethylated in III-1 than in III-2, while *H19/IGF2*, *DIRAS3*, *MEST*, *KCNQ1OT1*, *NHP2L1*, and *IGF1R* were more hypomethylated in III-2 than in III-1 (Fig. 2c, Additional file 4: Figure S3). Consistent with a role of *NLRP5* in post-zygotic imprinting maintenance, both maternally and paternally methylated gDMRs were hypomethylated in the two siblings. As expected, paternally methylated secondary DMRs (*GNAS-NESP*, *ZNF597*, and *ZDBF2/GPR1*) were hypermethylated if their respective maternally methylated gDMRs were hypomethylated. *KCNQ1OT1* and *IGF1R* were found slightly hypomethylated also in II-2 (Fig. 2c, Additional file 4: Figure S3). The milder MLID of this individual could be explained by the presence of only one *NLRP5* variant (R533C) in her mother (I-2, see Fig. 1d). Outside the imprinted loci, only a few other regions were found differentially methylated in these individuals relative to controls (Additional file 5: Table S2).

To compare the methylation profiles of this family with those of other MLID patients, we collected array data sets from three further studies [5, 6, 20]. These included patients presenting with the clinical features of BWS, SRS, TNDM, pseudohypoparathyroidism 1B (PHP1B), Temple syndrome (TS), or complex imprinting disorders. After adjusting for batch effects in the datasets (see the “Materials and methods” section), we investigated if any similarity could be identified with the imprinted DMR methylation profiles of our family. When DNA methylation at imprinted loci was analyzed by principal component analysis (PCA), control individuals clustered together and separately from MLID patients (Additional file 6: Figure S4a). However, no evident clustering was observed for the MLID profiles associated with BWS and other phenotypes. In particular, the profile of III-2 did not cluster with that of III-1, but it was not better separated than III-1 from the other BWS-MLID cases. Unsupervised hierarchical clustering of the methylation profiles also clearly separated the MLID cases from the controls, but again the individual clinical phenotypes associated with MLID were not clearly clustered (Additional file 6: Figure S4b). Variegated patterns of DMR methylation were also observed in the six individuals with maternal *NLRP5* variants (Additional file 6: Figure S4b; Additional file 7: Table S3). III-1 shared with some of the other *NLRP5*-MLID individuals his 22 dysregulated DMRs (both hypo- and hypermethylated regions out of 42 investigated), while III-2 shared 14 out of his 16 dysregulated DMRs with others. However, none of the DMRs was affected in all these individuals and only two DMRs (*KCNQ1OT1* and *IGF1R*) were hypomethylated in five of six of them, indicating that no specific DNA methylation signature could be associated neither with *NLRP5* variants nor with a specific clinical phenotype among MLID patients.

In this study, we show that MLID associated with maternal variants can be found in individuals with limited clinical features that are not enough for clinical diagnosis of imprinting disorder (e.g., III-2 has a BWS clinical score = 3) or other pathology. In addition, we confirm that molecular testing for BWS is indicated in the presence of a score of ≥ 2 to avoid the lack of follow-up of patients that do not meet the criteria for a clinical diagnosis [2]. Although MLID detection by genome-wide analysis is challenging and not suitable for routine testing, a MS-MLPA assay (ME034, MRC-Holland) that can detect MLID patterns is available and can be offered to patients with *KCNQ1OT1* LOM and a family history of BWS spectrum and no 11p15.5 CNV to determine if further testing for maternal variants should be considered [2].

In this family, we demonstrated two novel maternal-effect variants of *NLRP5* that further add to the genetic heterogeneity of MLID. Our findings confirm that the spectrum of phenotypes of the progeny of women with *NLRP5* variants is very wide, ranging from intrauterine death to apparently healthy phenotype and including different types of imprinting disorders.

The reason for this wide phenotypic heterogeneity is unknown. Our data indicate that the contrasting clinical phenotypes observed in our family were not due to dramatic differences in the extent of imprinted methylation, but possibly to subtle differences in the affected loci. Maternal *NLRP5* alterations may lead to mosaic methylation defects affecting a variable number of imprinted loci. This may explain the variegation of methylation patterns we observed in the affected individuals. The consistent pattern of gDMR methylation between the three tissues investigated may possibly reflect the early origin of the methylation defect, likely before implantation, in our family. It is possible that the complex clinical phenotype reflects the different loci affected. For example, the atypical features of III-1 may be caused by abnormal methylation of loci other than *KCNQ1OT1*. In particular, the low birth weight may result from *PLAGL1* hypomethylation (a hallmark of TNDM), hypocalcemia may derive from *GNAS* hypomethylation (hallmark of PHP1B), and the cognitive delay may be associated with *SNRPN* hypomethylation (a hallmark of Angelman syndrome). It may also be speculated that since some imprinted genes exert opposing influences on growth, both growth stimulation (e.g., that derived from *CDKN1C* downregulation) and growth inhibition (e.g., that derived from *H19* activation which may be expected from hypomethylation of its DMR) may be present, and a sort of compensation may occur in III-2, as well as in the individuals with healthy phenotype of other cohorts. Although we cannot exclude the presence of mild clinical features when she was younger, a similar situation may have occurred in II-2. However, we could not identify any correlation between epigenotype and phenotype among the patients with MLID nor among the ones with maternal *NLRP5* variants. The presence of modifier genes or the differential effect of the variants on protein activities may contribute to phenotype variegation. Further multi-center collaborative studies are therefore needed to address the important question of the effect of MLID on health.

## Materials and methods

### Study subjects and family history

The proband (III-1, Fig. 1a) is a 20-year-old boy of unrelated parents. He was born at the 39th week of gestation with a weight of 1840 g (< 0.4th centile) and a length of 43 cm (< 3rd centile). At birth, he presented with placentomegaly, macroglossia, bilateral anterior earlobe pits, facial naevus simplex (forehead and nape), hypertelorism, small mouth, and asymmetry of the chest. Hypoglycemia and hypocalcemia occurred in the perinatal period. Feeding difficulties and episodes of apnea because of the macroglossia were also reported. In the medical record, it is reported that macroglossia decreased over the years showing a slight asymmetry with the left side bigger than the right side. Hemihyperplasia of the face (left side > right side), upper limbs (left side > right side), and lower limbs (right side > left side) were also observed. Dysmetric right femur and tibia were surgically corrected when the proband was 10 years old. Clinical examination at 2 years revealed a slight language and learning delay. Subsequently, a mild cognitive retardation was confirmed.

His sibling (III-2) is a male of 11 years of age, born at the 36th week of gestation with placentomegaly but normal growth parameters. No clinical features of disease have been reported and currently, he shows only a slight facial asymmetry (left side > right side). Good school records are reported. According to the international consensus for BWS [2], a score = 3 that is not sufficient for clinical diagnosis but should be considered for molecular testing could be assigned to this case.

The proband’s mother (II-2) has a healthy phenotype apart from recurrent reproductive failures. She had four spontaneous pregnancy losses: the first at the 12th week of gestation; the second and third around the 23rd gestational week, both with evidences of placentomegaly; and the fourth miscarriage occurred at the 29th week of gestation and the fetus presented with exomphalos.

The proband’s father and maternal grand-parents showed healthy phenotypes. No mental retardation or further recurrent miscarriages or congenital malformations were reported in the family.

Sex and age information of the controls used in our study are reported in Additional file 8: Table S4. Information on the controls used in the study of Bens et al. [20] is reported in ref. [25]. In both studies, the controls are age-matched and composed of equal numbers of male and females.

### Ethics statement

The study was approved by the Ethical Committee of the University of Campania “Luigi Vanvitelli” (approval number 1135, 13 October 2016) and it was carried out according to the ethical principles and legislation of Italy. Written informed consent was obtained from the individuals involved in the study.

### Human samples

Genomic DNA was isolated from peripheral blood lymphocytes by using the salting-out procedure. The Puregene Buccal Cell Core kit (Qiagen, cat. n.158845) and Quick-DNA Urine kit (Zymo Research, cat. n. D3061) were used for the extraction of genomic DNA from buccal brush and urine, respectively, following the instructions of the manufacturers.

### Targeted DNA methylation analysis by pyrosequencing

Two micrograms of genomic DNA were treated with sodium bisulfite by using the EpiTect Bisulfite kit (Qiagen, cat. n. 59104) following the manufacturer’s protocol. About 200 ng of converted DNA was amplified by using the PyroMark PCR kit (Qiagen, cat. n. 978705) in a final volume of 25 μl and 10 μl of PCR product was used for quantitative DNA methylation by pyrosequencing on a Pyromark Q48 Autoprep system with the PyroMark Q48 Adv. CpG Reagents (Qiagen, cat. n. 974022) and PyroMark Q48 Magnetic Beads (Qiagen, cat. n. 974203). Results were analyzed by using the Pyromark Q48 Autoprep software. The Pyromark Assay Design SW 2.0 was used to design amplification and sequencing primers, listed in Additional file 9: Table S5. All primer sets used had a quality score assigned by the software > 70. The pyrosequencing approach has been used before as a method that quantitatively evaluates the methylation levels of imprinted regions [26, 27]. The specificity of the assay is ensured by the base-called sequence of the regions of interest given by the instrument and only CpGs with methylation level that passed the quality check were considered. Concerning sensitivity, the minimal detectable methylation level of each assay was determined by testing a mix of different ratios of methylated and unmethylated synthetic DNA (Zymo Research, cat. n. D5014) reproducing methylation levels between 1% and 100%. The minimal detectable range of methylation was estimated to be between 2% and 100% for all the five regions tested.

### Whole-exome sequencing

Whole-exome sequencing of parents and proband was performed on DNA derived from peripheral blood, and sequenced 100 bp paired-end at IGA Technology Services (Italy) using the Agilent SureSelect Human All Exone v5 (50M bp of genome) library and the Illumina HiSeq2500 platform.

Reads were aligned to the human genome reference assembly (Homo_sapiens_assembly38.fasta) using the BWAmem software package v0.7.15 [28]. PCR duplicates were filtered out by Picard v2.9 (http://picard.sourceforge.net) and the GATK v3.7 suite was used to locally realign around inferred Insertion/Deletions (InDels) and recalibrate base quality scores. Single-nucleotide variants and InDels were called using GATK HaplotypeCaller and GenotypeGVCFs [29] and recalibrated with VariantRecalibrator. Recalibrated variants were annotated using wANNOVAR [30]. Genome variants with low coverage (< 15) or low quality (< 20) or frequently occurring in general population (MAF > 0.01 in 1000 Genomes Project [31] or Exac (http://exac.broadinstitute.org/), [23] or gnomAD [32]) were filtered out.

### Sanger sequencing

Two pairs of primers were used for amplification and Sanger sequencing (Eurofins Genomics) of the novel variants in exon 7 of the *NLRP5* gene (Additional file 9: Table S5).

### High-resolution SNP-array analysis

High-resolution SNP-array analysis was carried out using the CytoScan HD array (Affymetrix, Santa Clara, CA, USA) as previously described [33]. Following this pipeline, any clinical relevant copy-number variations or uniparental disomy were detected in this family.

### Genome-wide DNA methylation array analysis

Peripheral blood DNA samples from the proband, his healthy sibling and parents, together with six control individuals, were assayed on the same methylation array. DNA was sodium bisulphite treated using the EZ DNA Methylation Kit (D5001, Zymo Research). Single-strand bisulfite converted DNA was quantified with the NanoPhotometer Pearl (Implen GmbH). Genome-wide methylation was performed on the Infinium MethylationEPIC 850 K Bead Chip (WG-317-1001, Illumina) using Illumina-supplied reagents and conditions. Fluorescence intensities were captured using Illumina HiScan SQ (Illumina). The array data was analyzed using R (v. 3.5.3). Beta-values were extracted from “idat” files by using the “Load” module of the “Champ” R package (v. 2.12.0) [34], with quality control options set as default. The quality control step retained 759,772 probes, which were used for further analysis. SWAN [35] normalization was applied, with the “method” option set to “minfi”. The SWAN normalized samples were assigned with respective genome coordinates based on probe name and manifest file (Illumina). We compared methylation profiles of imprinted DMRs by performing unsupervised clustering using beta-values of individual CpG probes present inside the coordinates of human imprinting control regions (ICRs) [24]. The average methylation profile for each DMR was determined by calculating the mean of beta-values of CpGs/probes present inside DMRs. DMRs overlapping less than 3 CpGs/probes were filtered out. We converted the ratios patient/controls to log-values and used for plotting. The plots were generated using *R* packages ggplot2 and heatmap. Heatmap represents the average beta-values of probes underlying coordinates of DMRs. The width of violin plots shows the density of probes carrying a range of methylation level. The box plot inside the violin plot depicts the interquartile range (25–75% probes) and a median line. To visualize the methylation levels, we tagged probe coordinates with the beta-values of each individual and uploaded to the UCSC genome browser. The raw and processed data are publicly available in the Gene Expression Omnibus (GEO) repository under accession number GSE133774.

Differentially methylated regions outside the imprinted regions were searched by using the Crawford-Howell (CH) *t* test [36]. First, we considered the methylation array probes present inside windows of 2-kb region of the human genome. We took the average of CpG methylation present in 2-kb bins and applied CH *t* test to calculate *p* value. We further performed multiplicity correction using the Benjamini-Hochberg method. Regions with *p*.adj value < 0.05 were considered with significantly altered methylation profile. Regions overlapping previously known human ICRs were marked as DMR and others were assigned as non-DMRs.

### Other datasets

To compare DNA methylation array data of our samples with other MLID cases and controls, we considered the shared CpGs probes (~ 386 K) of our dataset with the Infinium Human Methylation 450 K Beadchip (Illumina) array data from previous studies [5, 6, 20] (GEO: GSE78773). We used the combat function from sva R package (sva version 3.32.1) to adjust for batch effects in datasets (Additional file 10: Figure S5). The batch-adjusted matrix was used for further downstream analysis. We have also performed unsupervised clustering for individual CpG probes present inside imprinted DMRs, calculated the average methylation for each DMRs as described before, and normalized individual samples from different studies by the average of their respective controls. We converted the ratios patient/controls to log-values and used for plotting. Heatmap with hierarchical clustering (Ward’s method) and PCA plot were done considering only the shared CpG probes of imprinted loci between our and other datasets.

### Statistical analysis

The data was tested for statistical significance using Wilcoxon signed-rank test and two-tailed mode. Control samples were compared with every sample of the family (Additional file 3: Table S1).

## Supplementary information

**Additional file 1: Figure S1.** Methylation analysis of the *KCNQ1OT1*:TSS-DMR by COBRA. DNA methylation of the CpG included in the restriction enzyme site CCGG was assayed by COBRA in the proband and his parents. Bands corresponding to unmethylated and methylated DNAs are indicated at the right side of the panel and methylation levels for each individual are at the bottom.

**Additional file 2: Figure S2.** Analysis of *CDKN1C* expression level by quantitative RT-PCR. RNAs from oral mucosa have been tested in triplicate, in three independent experiments. Values were normalised against those of GAPD. P-value has been calculated by two-tailed Student’s T-test (*, P ≤ 0.05).

**Additional file 3: Table S1.** Statistical analysis.

**Additional file 4: Figure S3.** Examples of hypomethylated imprinted DMRs as visualized by UCSC genome browser. Each vertical line represents a CpG site. Asterisks indicate regions with discordant methylation levels in the two siblings. gDMRs: germline DMRs; sDMRs: secondary DMRs.

**Additional file 5: Table S2.** Differentially Methylated 2 kb regions.

**Additional file 6: Figure S4.** Methylation defects of imprinted DMRs in MLID cases. PCA plot (a) and of heatmap showing the result of unsupervised hierarchical clustering (b) of the CpG methylation values for 678 shared probes overlapped with 42 human imprinted DMRs from individuals with MLID as analyzed by HumanMethylationEPIC BeadChip (850 K) array (present study) and Infinium Human Methylation 450 K Beadchip array, normalized against their respective control individuals [5, 6, 20]. Maternally-methylated germline DMRs are in dark pink, maternally-methylated secondary DMRs in light pink, paternally-methylated germline DMRs in dark blue, paternally-methylated secondary DMRs in light blue. The cases with maternal-effect variants in *NLRP5* are highlighted in green.

**Additional file 7: Table S3.** Methylation levels of imprinted DMRs in individuals with maternal *NLRP5* variants.

**Additional file 8: Table S4.** Sex and age information of controls.

**Additional file 9: Table S5.** Primers utilized in this study.

**Additional file 10: Figure S5.** Batch effect adjustment of array datasets. PCA analysis of shared CpG probes (~386 K) before (a) and after (b) batch correction.

## Data Availability

Methylation array data generated and analyzed during the current study have been deposited under accession code GSE133774 in the Gene Expression Omnibus repository.

## Abbreviations

BWS: Beckwith-Wiedemann syndrome
BWSp: Beckwith-Wiedemann syndrome spectrum
CNV: Copy number variation
COBRA: Combined bisulfite restriction analysis
gDMR: Germline-derived differentially methylated region
IG-DMR: Intergenic-differentially methylated region
LOM: Loss of methylation
LRR: Leucine-reach repeat
MLID: Multi-locus imprinting disturbances
NOD: Nucleotide-binding and oligomerization domain
PBL: Peripheral blood leukocytes
PCA: Principal component analysis
PHP1B: Pseudohypoparathyroidism 1B
SCMC: Sub-cortical maternal complex
sDMR: Secondary differentially methylated region
SNP: Single-nucleotide polymorphism
SRS: Silver-Russell syndrome
TNDM: Transient neonatal diabetes mellitus
TS: Temple syndrome
TSS-DMR: Transcription start site-differentially methylated region
