# Supplementary figures and images for: The phenotypic variations of multi-locus imprinting disturbances associated with maternal-effect variants of NLRP5 range from overt imprinting disorder to apparently healthy phenotype
Source: Clin Epigenetics. 2019 Dec 11;11:190. doi: 10.1186/s13148-019-0760-8 (PMC6907351; doi:10.1186/s13148-019-0760-8)

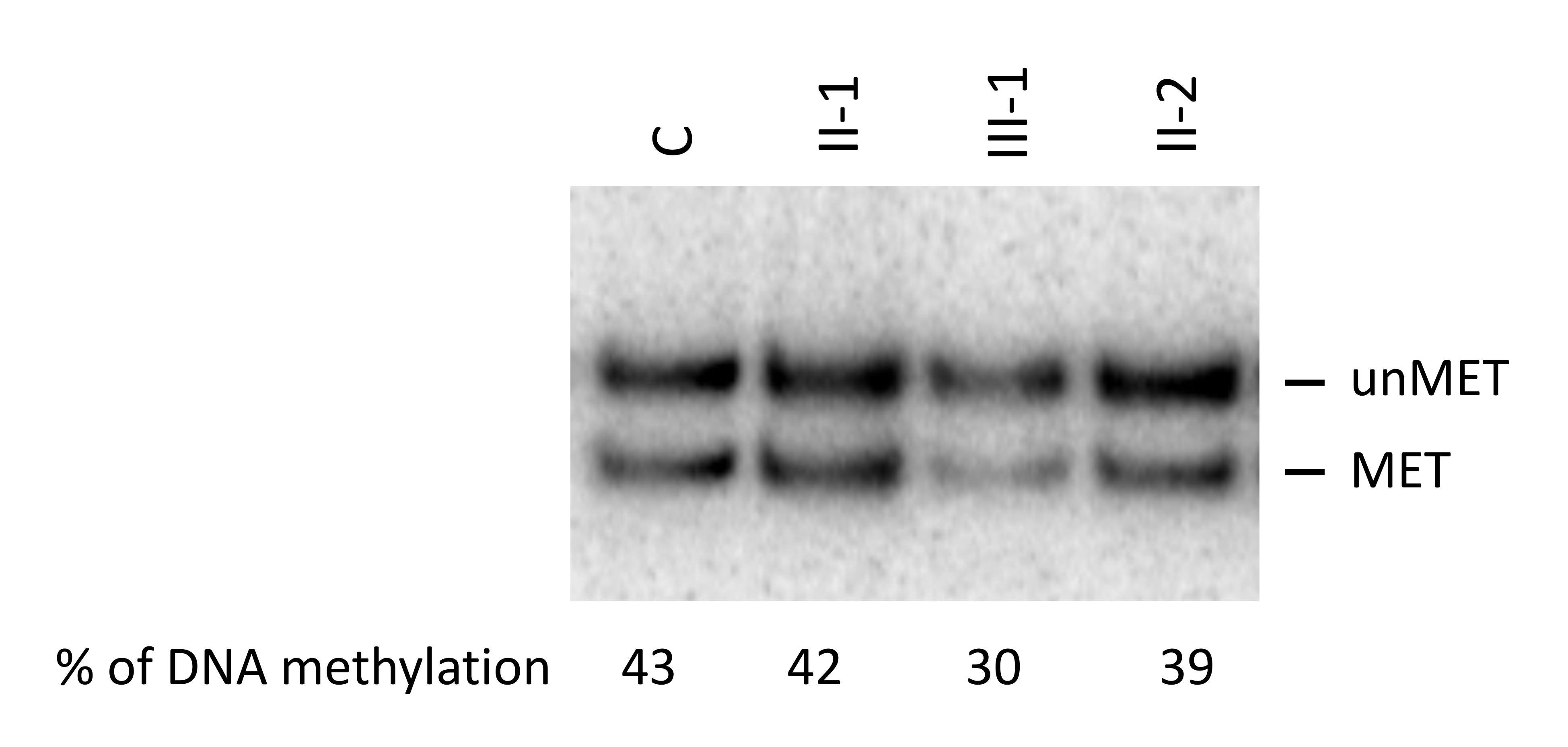

Supplement: Supplementary file 1 — Additional file 1: Figure S1. Methylation analysis of the KCNQ1OT1:TSS-DMR by COBRA. DNA methylation of the CpG included in the restriction enzyme site CCGG was assayed by COBRA in the proband and his parents. Bands corresponding to unmethylated and methylated DNAs are indicated at the right side of the panel and methylation levels for each individual are at the bottom. [file 13148_2019_760_MOESM1_ESM.tif]

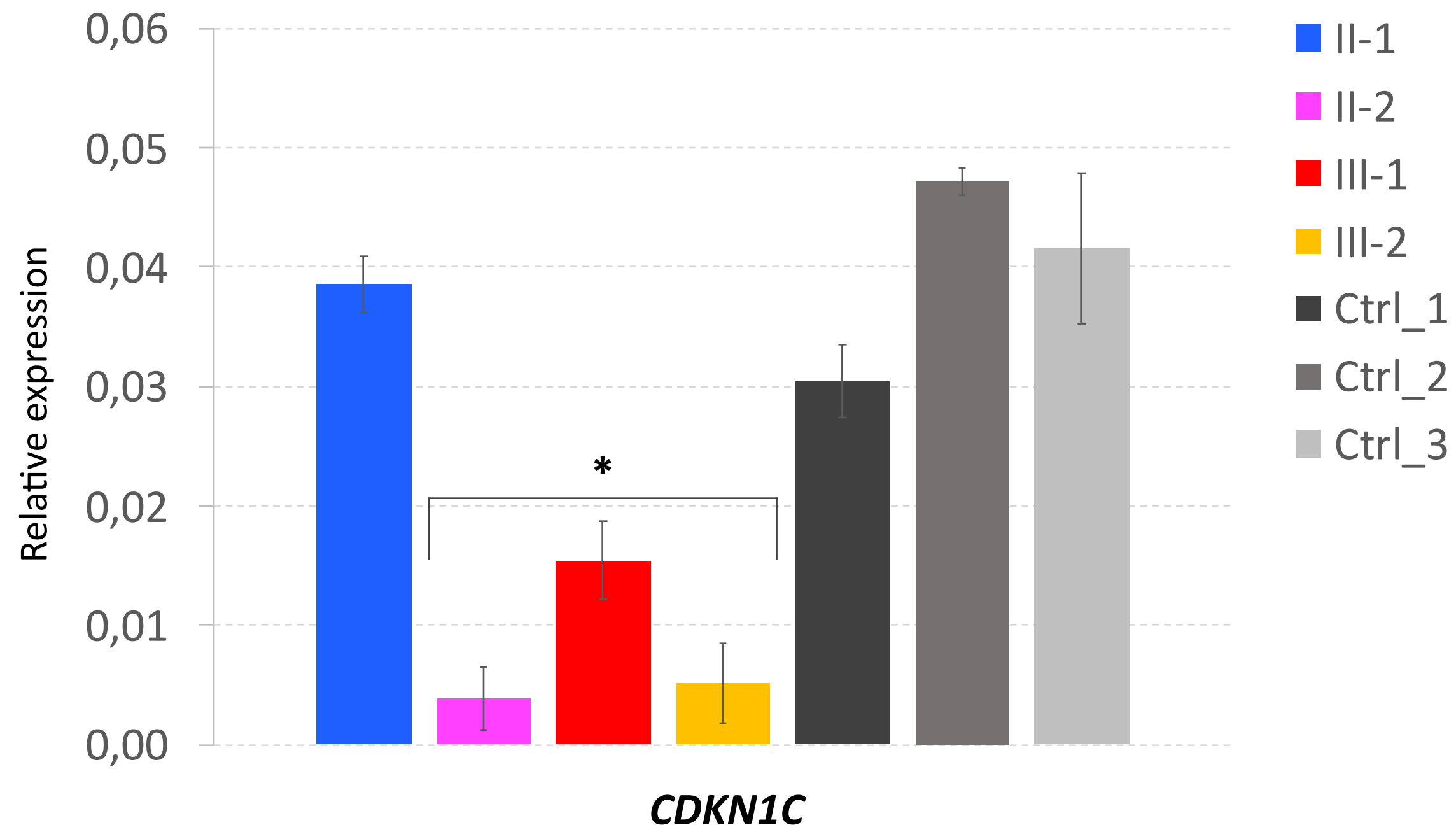

Supplement: Supplementary file 2 — Additional file 2: Figure S2. Analysis of CDKN1C expression level by quantitative RT-PCR. RNAs from oral mucosa have been tested in triplicate, in three independent experiments. Values were normalised against those of GAPD. P-value has been calculated by two-tailed Student’s T-test (*, P ≤ 0.05). [file 13148_2019_760_MOESM2_ESM.pdf]

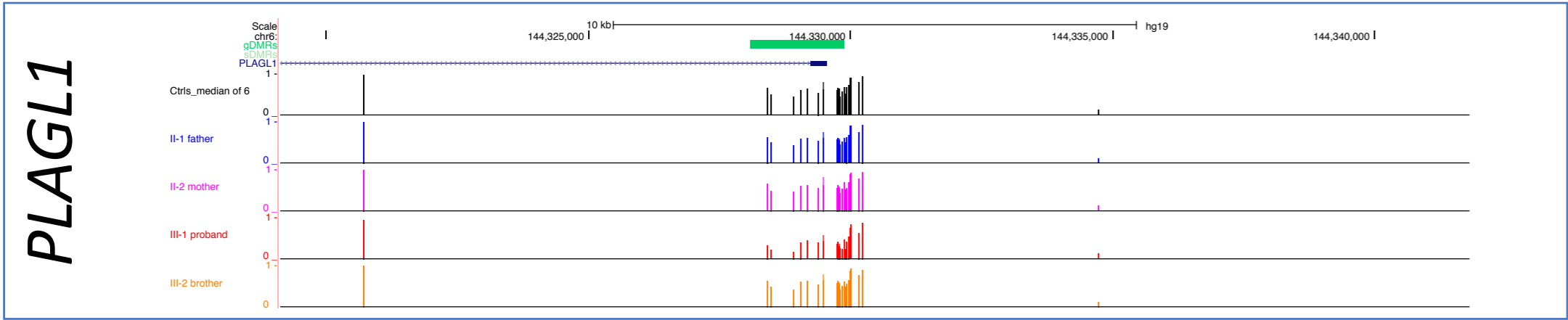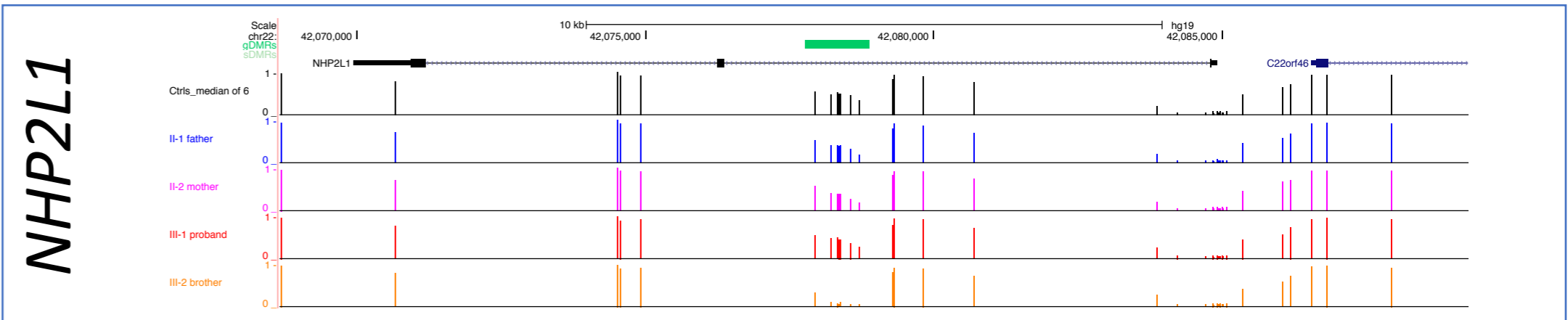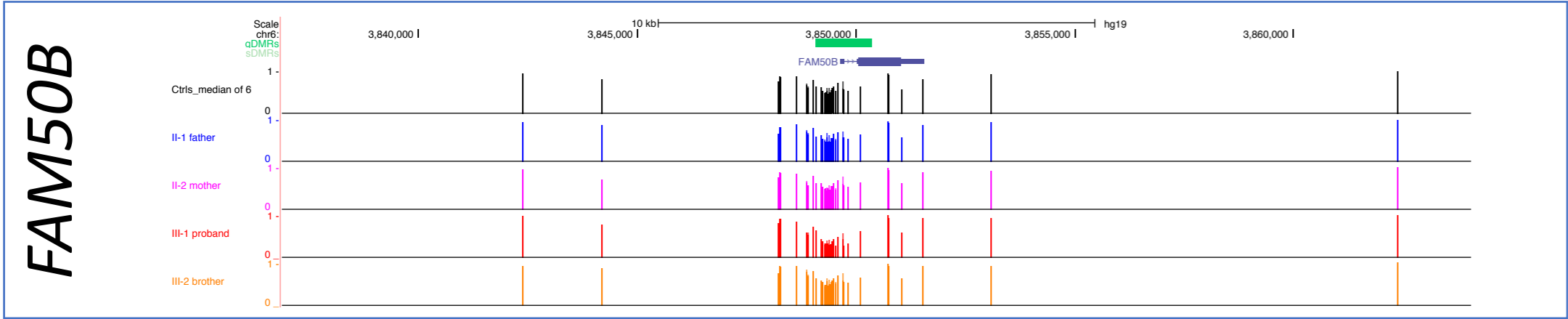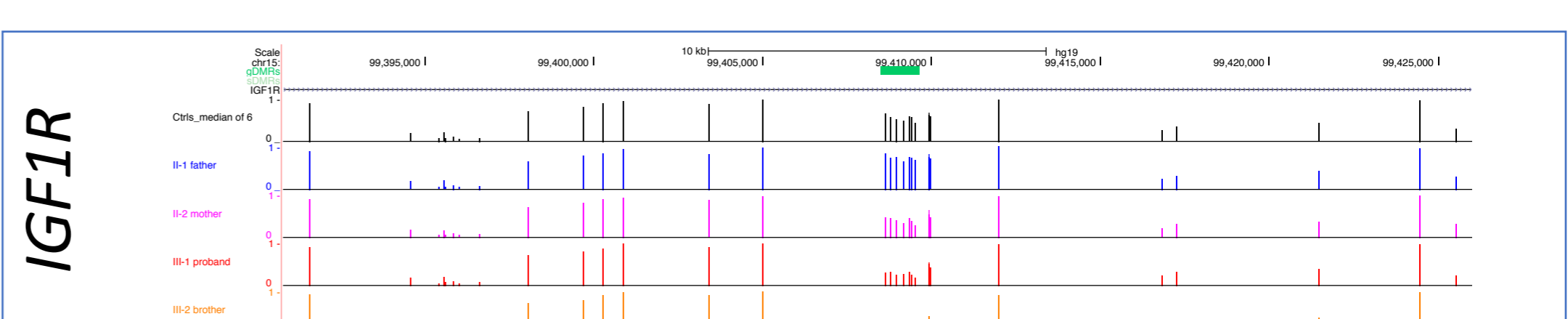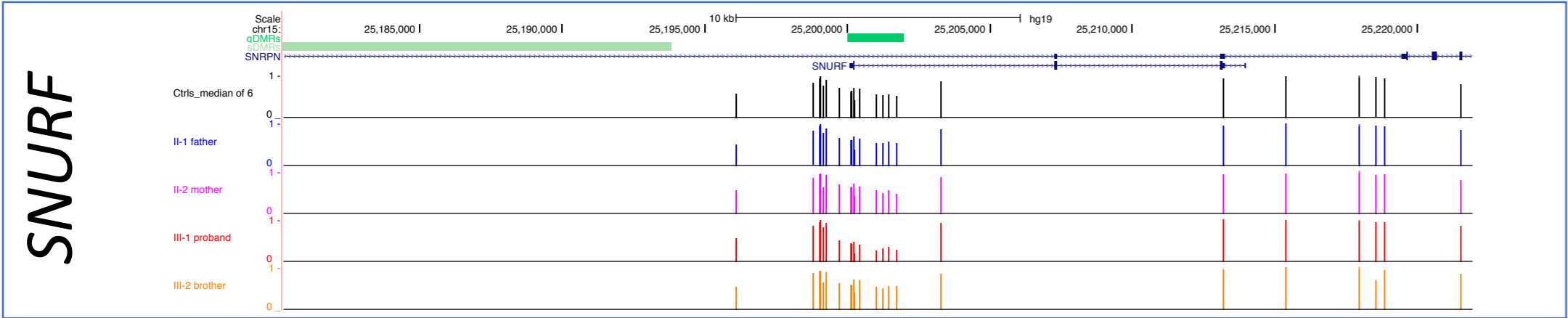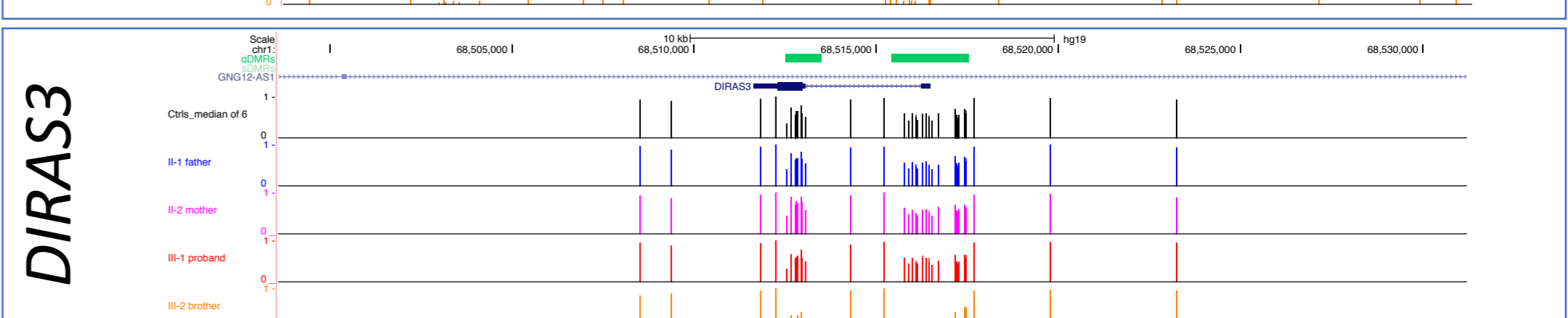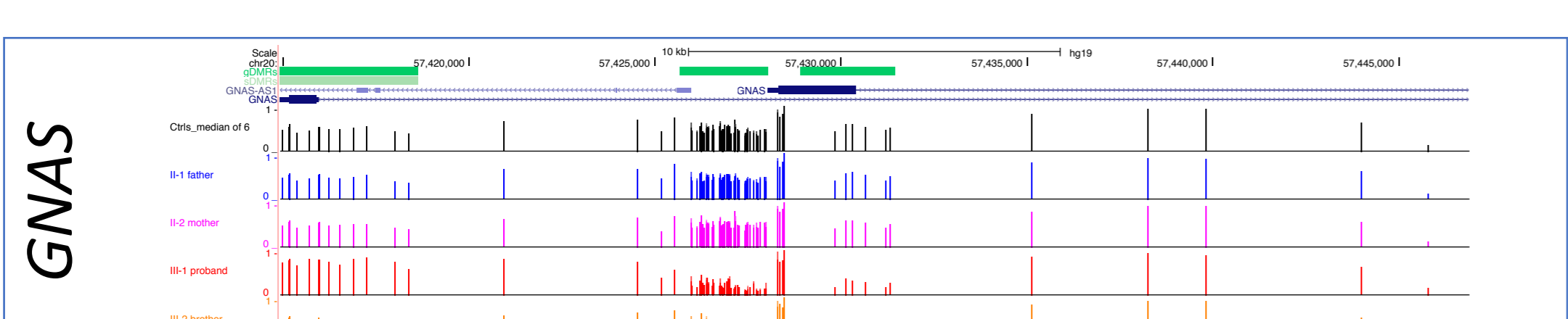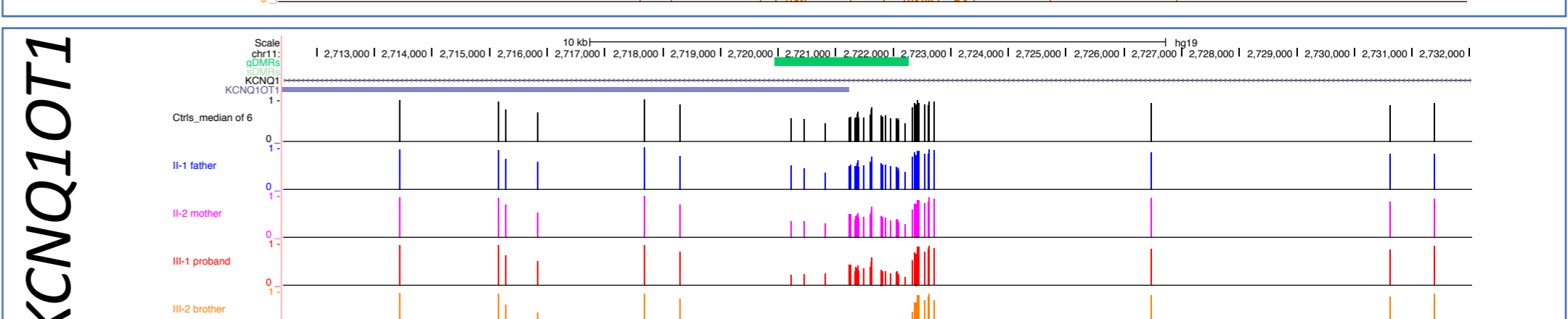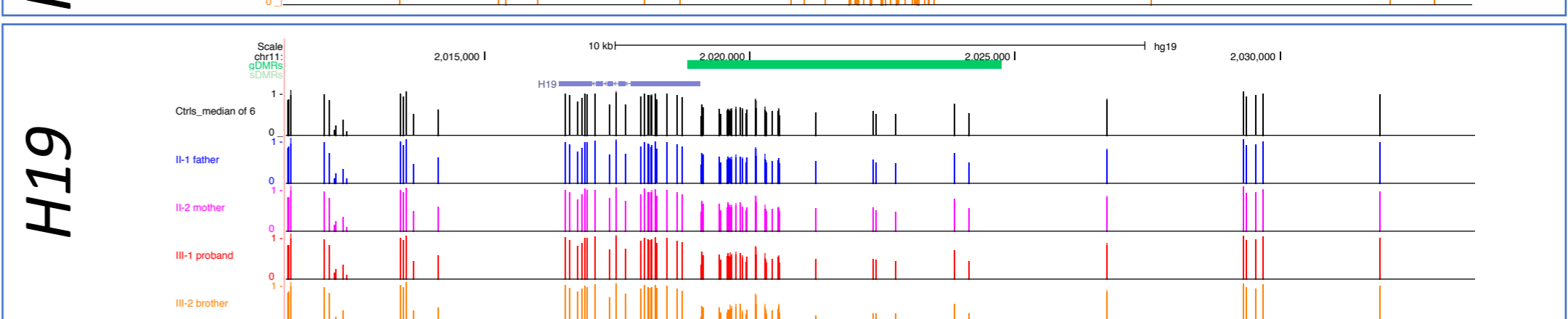

Supplement: Supplementary file 4 — Additional file 4: Figure S3. Examples of hypomethylated imprinted DMRs as visualized by UCSC genome browser. Each vertical line represents a CpG site. Asterisks indicate regions with discordant methylation levels in the two siblings. gDMRs: germline DMRs; sDMRs: secondary DMRs. [file 13148_2019_760_MOESM4_ESM.pdf]

a

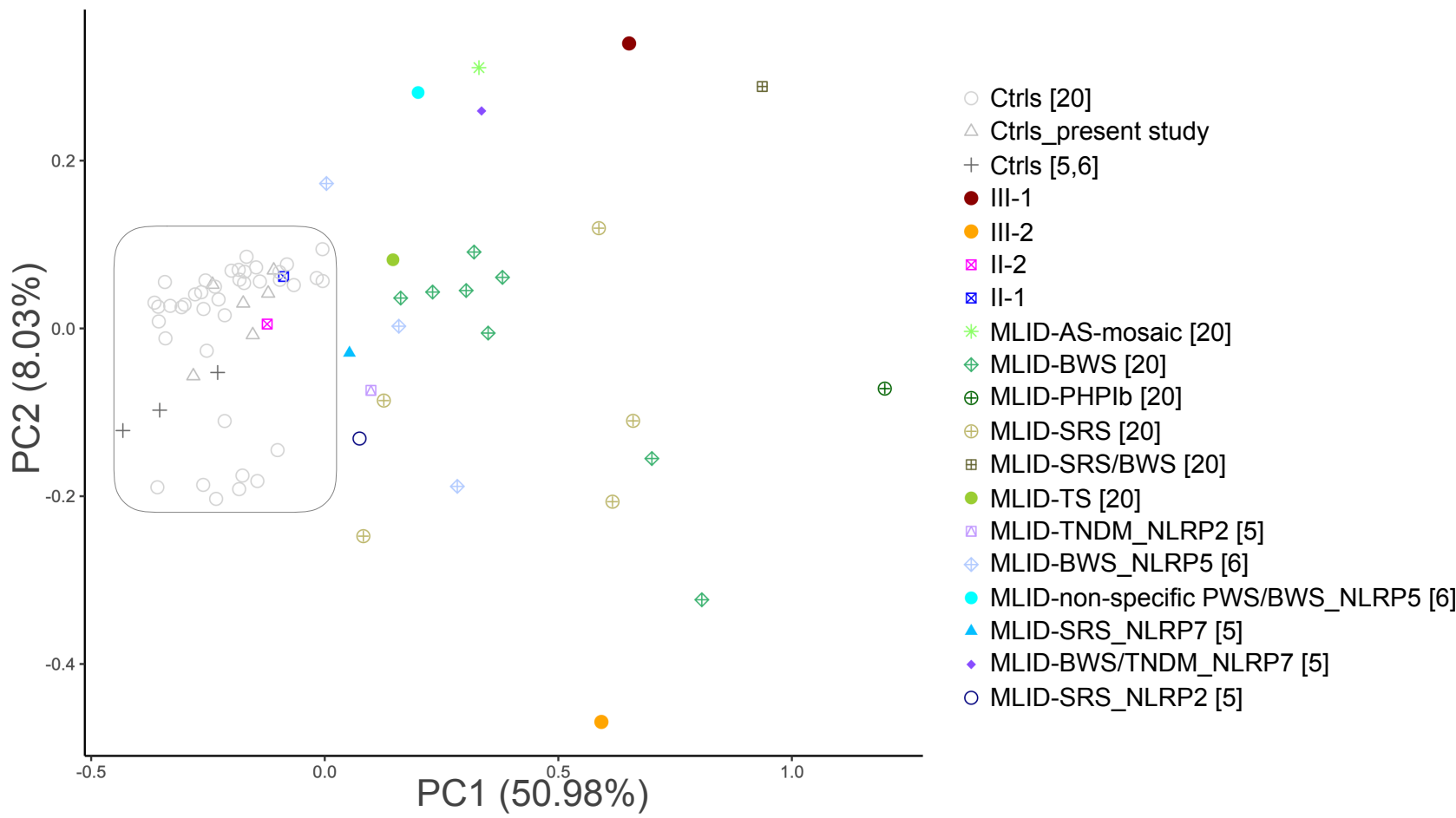

b

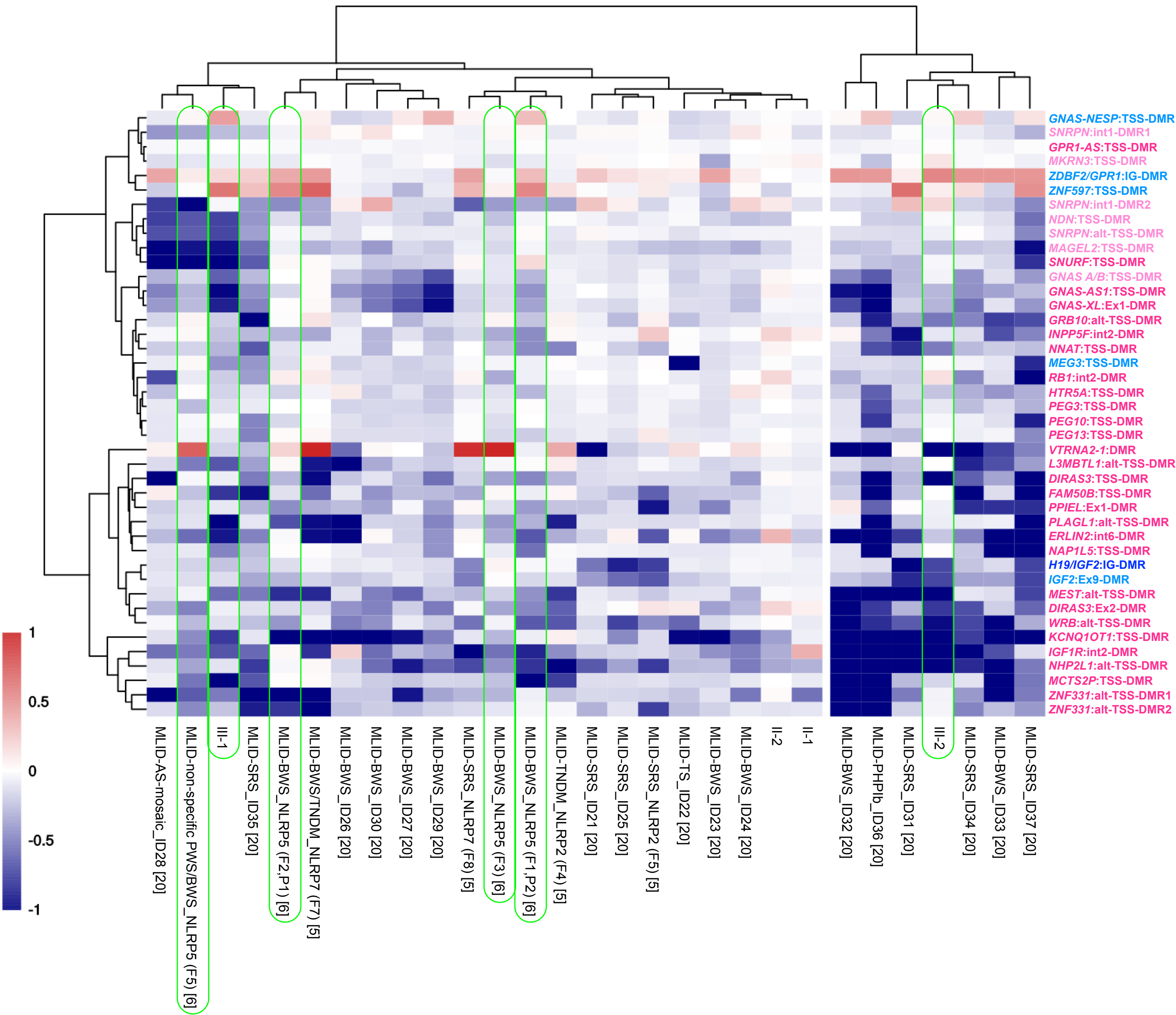

Supplement: Supplementary file 6 — Additional file 6: Figure S4. Methylation defects of imprinted DMRs in MLID cases. PCA plot (a) and of heatmap showing the result of unsupervised hierarchical clustering (b) of the CpG methylation values for 678 shared probes overlapped with 42 human imprinted DMRs from individuals with MLID as analyzed by HumanMethylationEPIC BeadChip (850 K) array (present study) and Infinium Human Methylation 450 K Beadchip array, normalized against their respective control individuals [5, 6, 20]. Maternally-methylated germline DMRs are in dark pink, maternally-methylated secondary DMRs in light pink, paternally-methylated germline DMRs in dark blue, paternally-methylated secondary DMRs in light blue. The cases with maternal-effect variants in NLRP5 are highlighted in green. [file 13148_2019_760_MOESM6_ESM.pdf]

**a****Uncorrected batch**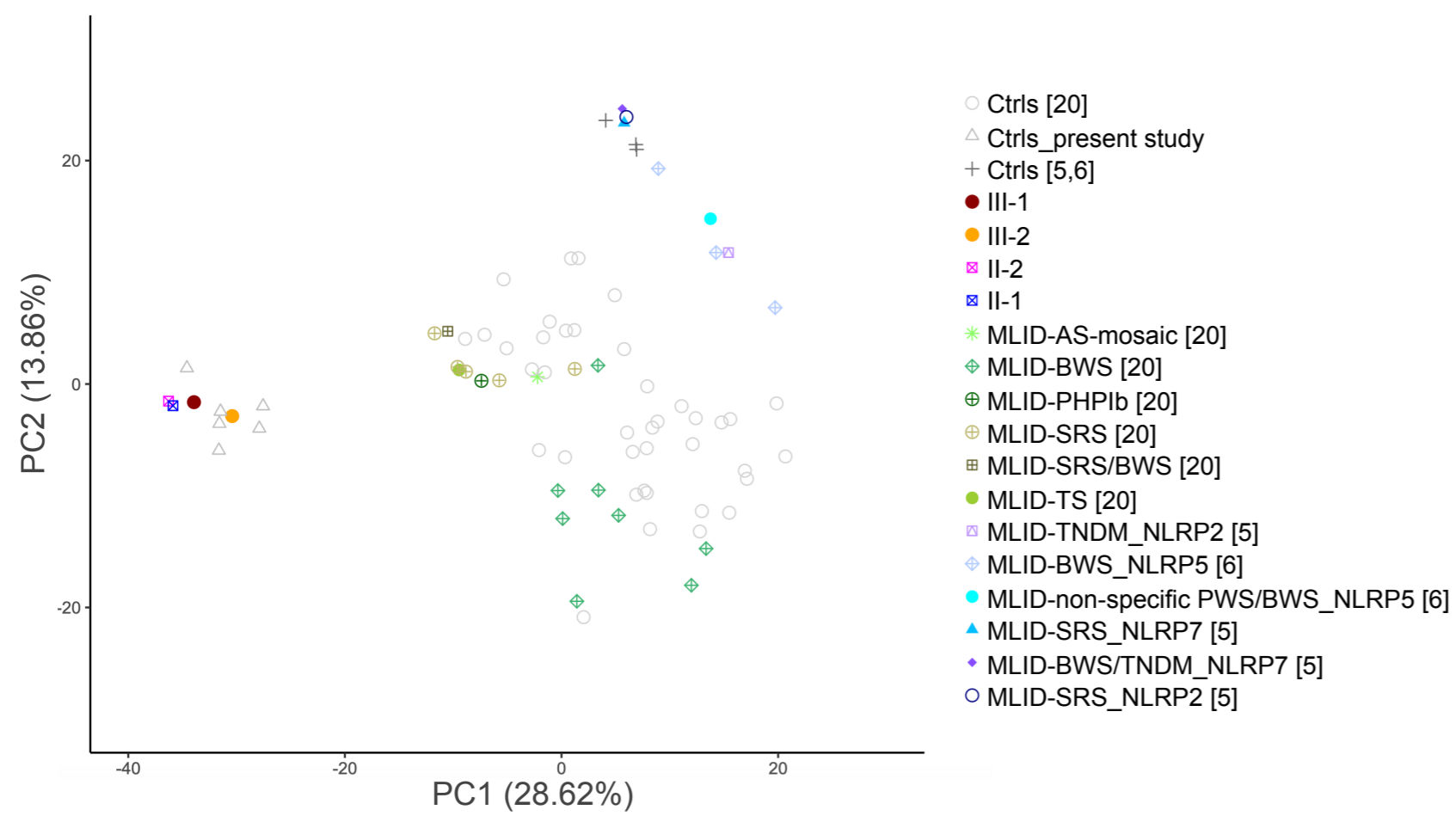**b****Batch effect adjusted**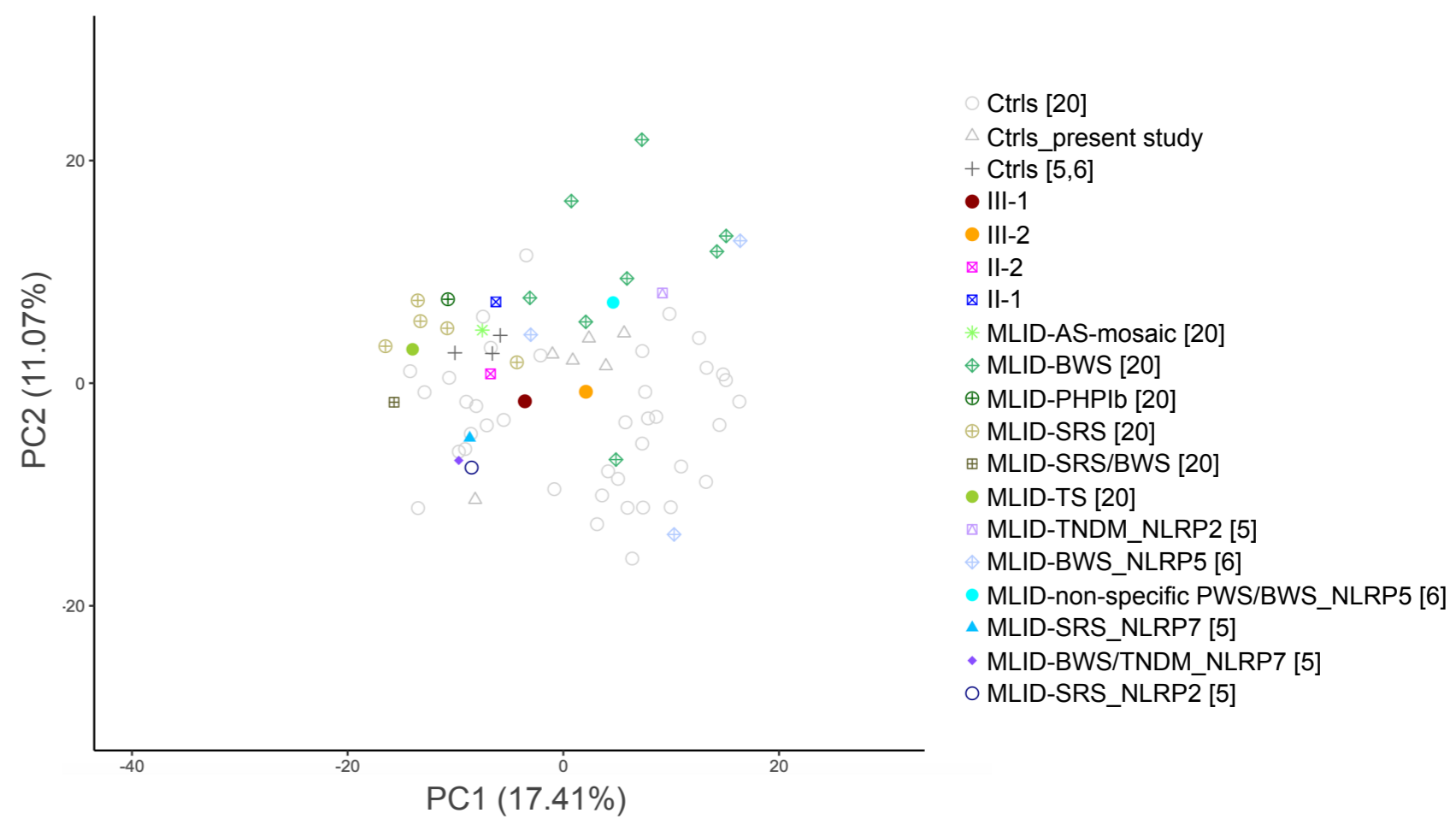

Supplement: Supplementary file 10 — Additional file 10: Figure S5. Batch effect adjustment of array datasets. PCA analysis of shared CpG probes (~386 K) before (a) and after (b) batch correction. [file 13148_2019_760_MOESM10_ESM.pdf]
